# Electrophysiological Signature and the Prediction of Deep Brain Stimulation Withdrawal and Insertion Effects

**DOI:** 10.3389/fneur.2021.754701

**Published:** 2021-11-30

**Authors:** Carlos Trenado, Laura Cif, Nicole Pedroarena-Leal, Diane Ruge

**Affiliations:** ^1^Laboratoire de Recherche en Neurosciences Cliniques, LRENC, Montpellier, France; ^2^Département de Neurochirurgie, Centre Hospitalier Universitaire de Montpellier, Montpellier, France

**Keywords:** electrophysiological signature, deep brain stimulation, dependency, neuropsychiatric disease, neuromodulation

## Abstract

Deep brain stimulation (DBS) serves as a treatment for neurological and psychiatric disorders, such as Parkinson's disease (PD), essential tremor, dystonia, Tourette Syndrome (GTS), Huntington's disease, and obsessive-compulsive disorder (OCD). There is broad experience with the short-term effects of DBS in individual diseases and their signs/symptoms. However, even in acute treatment and for the same disorder or a given disorder, a prediction of effect is not perfect. Even further, the factors that influence the long-term effect of DBS and its withdrawal are hardly characterized. In this work, we aim to shed light on an important topic, the question of “DBS dependency.” To address this, we make use of the Kuramoto model of phase synchronization (oscillation feature) endowed with neuroplasticity to study the effects of DBS under successive withdrawals and renewals of neuromodulation as well as influence of treatment duration in *de novo* DBS “patients.” The results of our simulation show that the characteristics of neuroplasticity have a profound effect on the stability and mutability of oscillation synchronization patterns across successive withdrawal and renewal of DBS in chronic “patients” and also in *de novo* DBS “patients” with varying duration of treatment (here referred to as the “number of iterations”). Importantly, the results demonstrate the strong effect of the individual neuroplasticity makeup on the behavior of synchrony of oscillatory activity that promotes certain disorder/disease states or symptoms. The effect of DBS-mediated neuromodulation and withdrawal is highly dependent on the makeup of the neuroplastic signature of a disorder or an individual.

## Introduction

Deep brain stimulation (DBS) is a neuromodulation technique that is effective as a treatment for severe neurological and psychiatric disorders (1–4). It has been suggested that it modulates cortico-striatal brain circuitry with an indirect effect on cognitive and behavioral abilities (5, 6). The overall efficacy of DBS for different pathologies, such as Parkinson's disease (PD) and dystonia, has been well-established for months and a number of years. The long-term effects remain overall effective on motor symptoms and mood (7, 8); they have, however, to be characterized further in terms of variability in efficacy and clinical adverse features, such as stimulation-related side effects or stimulation-independent effects. To further the demographic and history of disease-related predictors, understanding of relevant prediction factors for short- and long-term effects of DBS is required. Long-term neuromodulation with deep brain stimulation reorganizes the brain, changes the inherent patterns of cortical excitability typical of a particular disorder or symptom, and causes different individual clinical responses of a patient upon withdrawal of the stimulation input. One meaningful marker for the clinical withdrawal effect seems to be neuroplasticity which is quantifiable with electrophysiological recordings (9–12). Importantly, in patients *in vivo* it would be impossible to characterize the complex multifactorial patterns of neuroplasticity in a specific state (e.g., “ON DBS,” “OFF DBS,” and “symptom status”) the patient is in. The reasons for this are technical in nature. This explains the high value of a computational simulation as used here. It allows consideration of an input as a function of complex patterns, supposedly reflecting an electrophysiological signature of a patient.

A mechanism by which complex systems reach a specific state is synchronization of the lower-level elements that are organized into a functional unit (13). Synchronization has been referred to as the property of a non-linear system in which the dynamics of individual elements are correlated in time (14). Computational models have shown that synchronized spiking within small neural populations in cortical and hippocampal areas may be enhanced through Hebbian learning, which is characterized by long-term potentiation (LTP) if a presynaptic spike precedes a postsynaptic spike within a brief time window or by long-term depression (LTD) if the temporal order of spikes is reversed, a relationship described as *neurons that fire together wire together* (15–18). The invasive and non-invasive brain stimulation approaches allow a quantification of synaptic strength in the human nervous system, and manipulation of it has implications for the treatment of neurological and psychiatric disorders (19, 20). Exaggerated oscillatory neuronal synchronization relates to the cardinal symptoms of bradykinesia, rigidity, dystonia, and levodopa-induced dyskinesias. Excessive theta synchronization is a finding in dystonia, sensorimotor integration, and motor learning (21–25). Excessive beta oscillations have often been linked to specific Parkinson symptoms. It has been hypothesized that DBS is able to interrupt pathological synchronization (26).

At large-scale levels, for instance by considering electroencephalography (EEG) and magnetoencephalography (MEG) data, a valid index of synchronization is in-phase activation of neural elements in relation to cognition and pathology. In particular, alpha-beta phase synchronization has been reported to mediate the recruitment of visuospatial attention (27), while the role of selective attention in controlling phase oscillatory neural activity to efficiently process relevant information at pre-stimulus stages has been emphasized (28). In addition, frontotemporal theta phase-synchronization has been shown to underlie music-evoked pleasantness (29), while inter-brain phase synchronization has been proposed to be a marker of human social interaction (30). With regards to pathology, previous studies reported that deficits in EEG phase synchrony may underlie cognitive disturbances in schizophrenia (31) and that aberrant multi-frequency MEG phase temporal synchronization may be useful to predict conversion from mild cognitive impairment to Alzheimer's disease (AD) (32). Likewise, EEG phase synchrony has been helpful to prognosticate the outcomes in pediatric coma (33). Mean phase coupling of the motor brain regions has been shown to be abnormally enhanced in patients with PD and isolated dystonia (34, 35). In contrast, pianists with musician's dystonia exhibited deficient phase coupling between the neuronal assemblies required to inhibit motor memory traces (36). Increased EEG phase synchronization in all bands has been shown to be present in patients with Huntington's disease and to correlate with cognitive decline (37). The enhanced coupling or synchronization seems to be a feature of pathology, e.g., rigidity in PD or dystonic symptoms in dystonia. The desynchronization (or decoupling) on the other hand rather reflects “leaving of (also pathological) state,” in line with Pfurtscheller's work (38), while synchronization in the oscillations has a physiological healthy function (e.g., idling states) and the lack of mutability (change between states) bears pathology. For clinicians who use DBS as a treatment, pressing questions are: “What happens with the patient when I start DBS, when I switch off DBS after a short treatment duration, after a long treatment duration, when it accidentally stops working”? And moreover, “Is the patient dependent on the DBS or is there a window in time where I can ultimately stop DBS and the patient reaches independence?”

In the present study, we made use of computational modeling using an established network's model of synchronization, Kuramoto's model, endowed with plasticity (39). First, we targeted the effect of consecutive withdrawals and renewals of DBS by considering the different neuroplasticity conditions defined by the levels of (long term) potentiation and depotentiation. Second, we examined the effect of stimulation duration (by varying the number of iterations in the model) in *de novo* DBS “patients,” again under different neuroplasticity conditions. The results of our computer simulation mirror relevant clinical observations and also broaden our understanding of the long-term effect of DBS under the considered conditions.

## Materials and Methods

### Kuramoto's Model With Endowed Plasticity

As emphasized by previous DBS studies, the state of a neuron can be described by a set of variables that for certain parameters display a regular behavior. Therefore, such a state is susceptible to be described by the parameters that reflect changes in regularity as in the case of the phase (40). The same notion naturally applies to the ensembles of neurons whose regular behavior gives place to the patterns of regularity that have been linked to high cognitive functions and behavioral features as described earlier. On the basis of such an observation, we adopt Kuramoto's model of network synchronization to address the long-term effects of DBS.

In accordance with Kuramoto's model, the phase evolution equation for a network of coupled oscillators is given by:


$$\begin{array}{l} \frac{\partial }{\partial t}\varphi_{i}=\omega_{i}+\frac{K_{ij}}{N}\overset{N}{\underset{i=1}{\sum }}\text{sin}\left(\varphi_{i}-\varphi_{j}\right)+I_{i}, \end{array}$$

where φ_*i*_ and ω_*i*_ represent the phase and natural frequency of oscillator *i*, *K*_*ij*_ refers to the coupling between the oscillators *i* and *j*, *N* represents the number of oscillators (*N* = 100), and *I*_*i*_ denotes the DBS input received by the oscillator *i*. The values for ω_*i*_ were uniformly distributed random numbers in the interval (0,1). The DBS input adopted was a stereotypical train of rectangular pulses with a 130 Hz frequency and a 3.0 amplitude. Note that a modified version of this model has been previously used to evaluate the efficacy of new therapeutic DBS protocols (40). As in previous studies (39), we assume a direct effect of plasticity on the coupling between the oscillators as defined by:


$$\begin{array}{l} K_{ij}=a_{p}⋆exp\left(\frac{r_{1}}{\tau_{p}}\right)-\alpha_{d}\;exp\left(\frac{r_{2}}{\tau_{d}}\right), \end{array}$$

where α_*p*_ and α_*d*_ refer to the potentiation and depotentiation rates, τ_*p*_ and τ_*d*_ denote the damping parameters (set-up as 0.5), and *r*_1_ and *r*_2_ (*r*_1_ ≠ *r*_2_) denote constant parameters that were selected from the uniformly distributed random values in the interval (0,1) for each pair of oscillators *i*, *j*.

### Synchronization Quantification

To quantify global synchronization for the considered network of coupled oscillators, we make use of the phase locking value (PLV) (41), which provides a normalized synchronization index [ranging from 0 (no synchronization) to 1 (full synchronization)] between a pair of oscillators *i*, *j* = 1.0.100 as defined by:


$$\begin{array}{l} PLV_{ij}=\frac{1}{N}\left|\overset{N}{\underset{i=1}{\sum }}e^{-i\left(\varphi_{i}-\varphi_{j}\right)}\right|, \end{array}$$

where φ_*i*_ and φ_*j*_ denote the phase of oscillators *i* and *j*. The grand average of *PLV*_*ij*_ across all possible combinations of pairs of oscillators (*i* ≠ *j* and without repetition) represents a global index of network synchronization for a given plasticity and DBS condition.

### Plasticity Conditions

In the present study, we considered different plasticity conditions on the basis of previous studies addressing the assessment of neuroplasticity in the case of patients suffering from neurodegenerative and psychiatric disorders as well as healthy subjects. For instance, a reduction of neuroplasticity as reflected in decreased LTP and LTD has been suggested in subjects with depression by transcranial magnetic stimulation (TMS) studies utilizing paired associative stimulation (PAS) (42). A deficient plasticity that is reflected in strong asymmetry of LTP and LTD has been suggested in patients suffering from bipolar disorder by studies targeting the effect of lithium on human plasticity (43). With regards to PD, lack of LTP in the primary motor cortex has been stressed by TMS studies utilizing intermittent theta burst stimulation (iTBS) (44). An excess of LTP has been suggested in the case of patients with dystonia (45).

Note that the plasticity conditions in the adopted synchronization model are defined by setting up the specific values for the potentiation and depotentiation rate parameters; specifically the following plasticity conditions were considered:

(1) high level of potentiation (α_*p*_ = 8.0) and low level of depotentiation (α_*d*_ = 0.001); (2) low level of potentiation (α_*p*_ = 0.001) and high level of depotentiation (α_*d*_ = 8.0); (3) equally high level of potentiation (α_*p*_ = 8.0) and depotentiation (α_*d*_ = 8.0); (4) equally medium level of potentiation (α_*p*_ = 4.0) and depotentiation (α_*d*_ = 4.0); equally low level of potentiation and depotentiation: (5) (α_*p*_ = 0.7) and (α_*d*_ = 0.7); (6) (α_*p*_ = 0.1) and (α_*d*_ = 0.1); and (7) (α_*p*_ = 0.001) and (α_*d*_ = 0.001).

### DBS Conditions

Focusing on the effect of consecutive withdrawal and renewal of DBS, the conditions DBS ON, DBS OFF, DBS ON2, DBS OFF2, and DBS ON3 were considered. For these conditions, duration and absence of DBS were set up to 2,000 iterations.

In the case of *de novo* DBS “patients,” the conditions DBS OFF and DBS ON were considered. For these conditions, duration, and absence of DBS stimulation were set up to 500, 1,000, and 2,000 iterations.

### Synchronization Percentage Change

For the long-term scenario of consecutive withdrawal and renewal of DBS, the condition DBS ON was defined as the baseline level (100%) so that percentage change in synchronization for the subsequent DBS conditions (DBS OFF, DBS ON2, DBS OFF2, and DBS ON3) was calculated in relation to DBS ON. Analogously, the initial condition DBS OFF was adopted as the baseline level (100%) in the case of *de novo* DBS “patients.”

## Results

**Figure 1:**
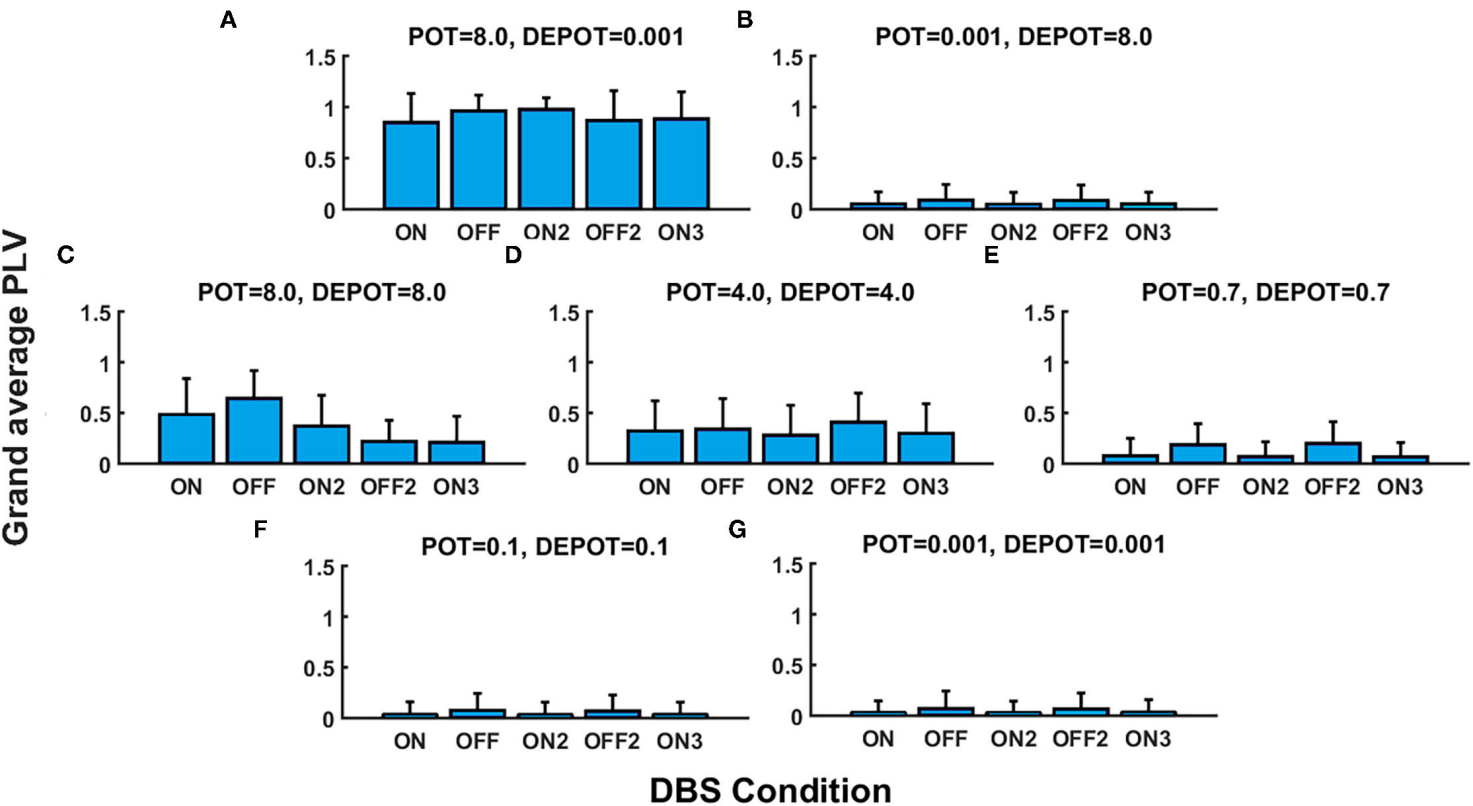
The simulation results (mean and SD of the grand average phase locking value [PLV]) corresponding to the long-term effect of deep brain stimulation (DBS) under successive withdrawal and renewal of stimulation: unbalanced potentiation and depotentiation (high and low level) **(A,B)**; balance potentiation and depotentiation, i.e., equally high levels, **(C)**; equally medium levels **(D)**; equally low levels **(E–G)**.

**Figure 2:**
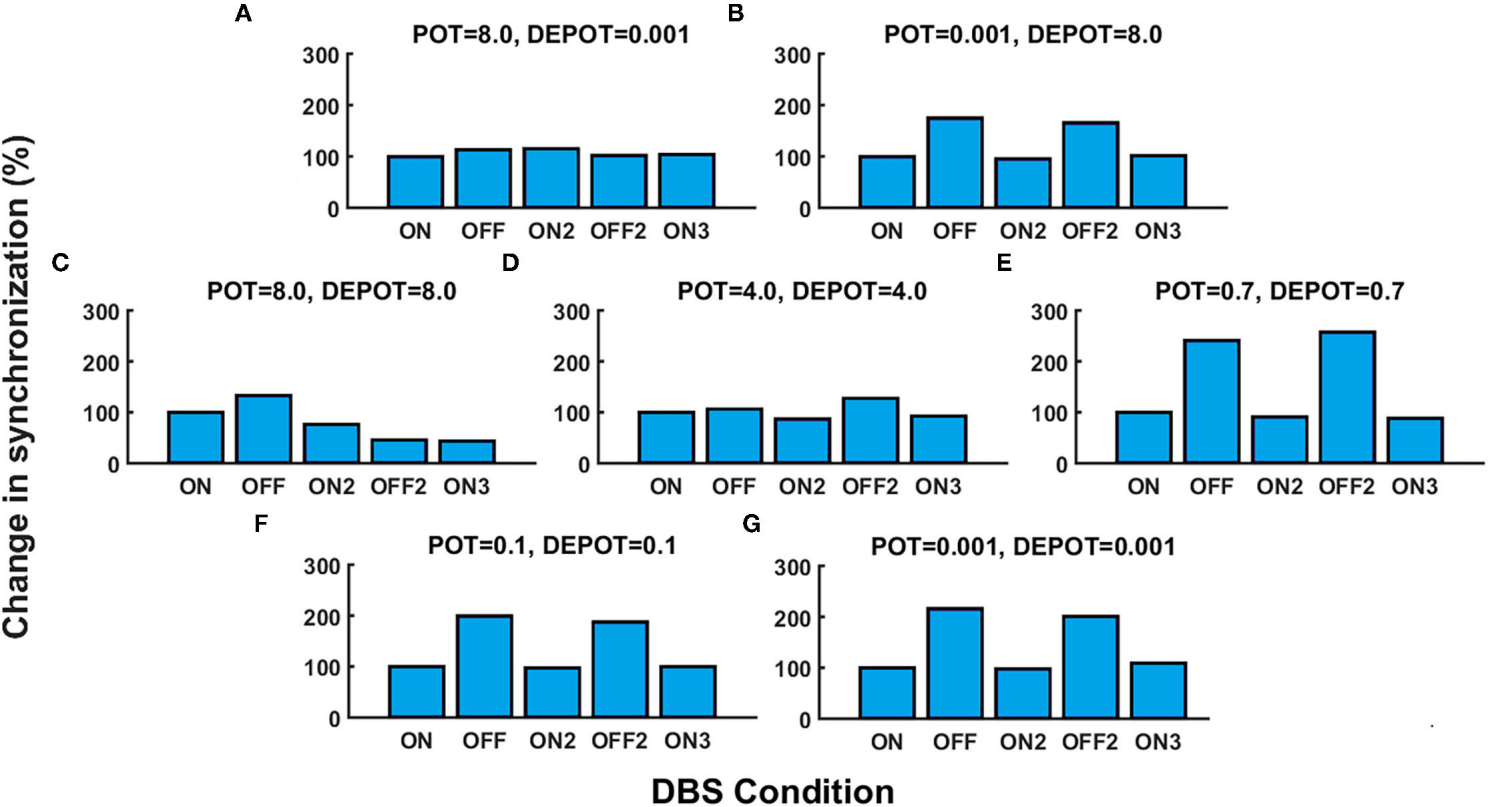
The simulation results [percentage change of the grand average PLV, DBS ON represents the baseline level (100%)] corresponding to the long-term effect of DBS under successive withdrawal and renewal of stimulation: unbalanced potentiation and depotentiation (high and low level) **(A,B)**; balance potentiation and depotentiation, i.e., equally high levels, **(C)**; equally medium levels **(D)**; equally low levels **(E–G)**.

With a focus on the long-term effect of DBS under successive withdrawal and renewal of stimulation, we varied the level and balance of potentiation and depotentiation as depicted in Figures 1A,B, 2A,B. Strikingly, a stable high phase locking value was noticeable in the case of high potentiation and low depotentiation (Figures 1A, 2A), whereas PLV fluctuated between increased and decreased very low values. There is a stable increase of PLV during the successive DBS OFF states in the opposite constellation of potentiation and depotentiation (Figures 1B, 2B). The case of symmetry (i.e., equally high, medium, or low) in the level of potentiation and depotentiation is depicted by Figures 1C–G, 2C–G. It is noticeable that for the high levels of potentiation and depotentiation (8.0 and 8.0), PLV tended to decrease under successive DBS withdrawal and renewals (Figures 1C, 2C); in the case of middle levels of potentiation and depotentiation (4.0 and 4.0), PLV showed a stable trend without fluctuations (Figures 1D, 2D); in the case of low values of potentiation and depotentiation (0.7 and 0.7), PLV fluctuated between increased and decreased low values with a consistent increase during the successive DBS OFF states (Figures 1E, 2E). In the case of very low levels of potentiation and depotentiation (0.1 as well as 0.001), PLV fluctuated between increased and decreased very low values with a stable increase during the successive DBS OFF states (Figures 1F,G, 2F,G).

**Figure 3:**
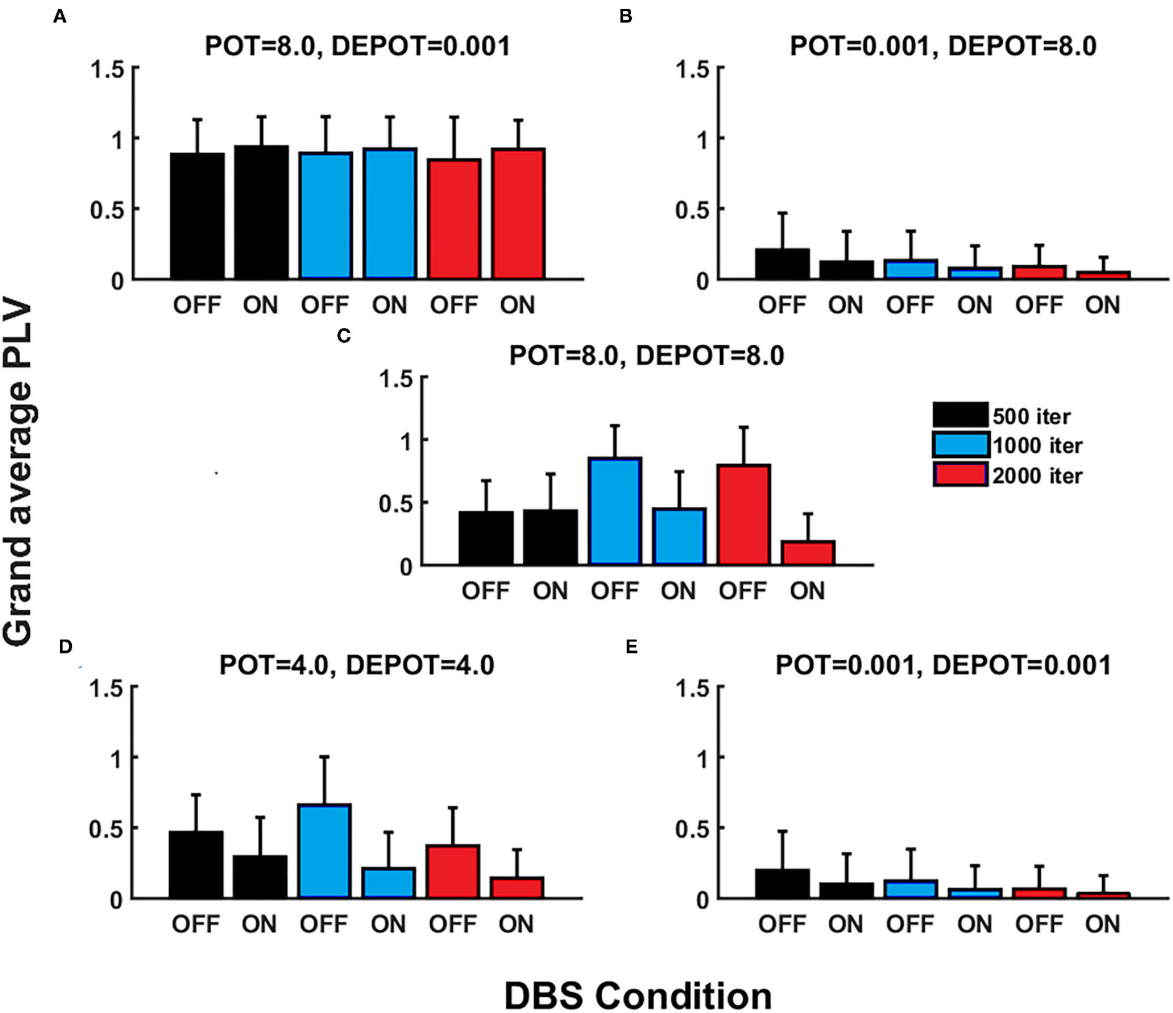
The simulation results (mean and SD of the grand average PLV) corresponding to the influence of stimulation duration (500, 1,000, and 2,000 iterations) in *de novo* DBS “patients”: unbalanced potentiation and depotentiation (high and low level) **(A,B)**; balance potentiation and depotentiation, i.e., equally high levels, **(C)**; equally medium levels **(D)**; and equally low levels **(E)**.

**Figure 4:**
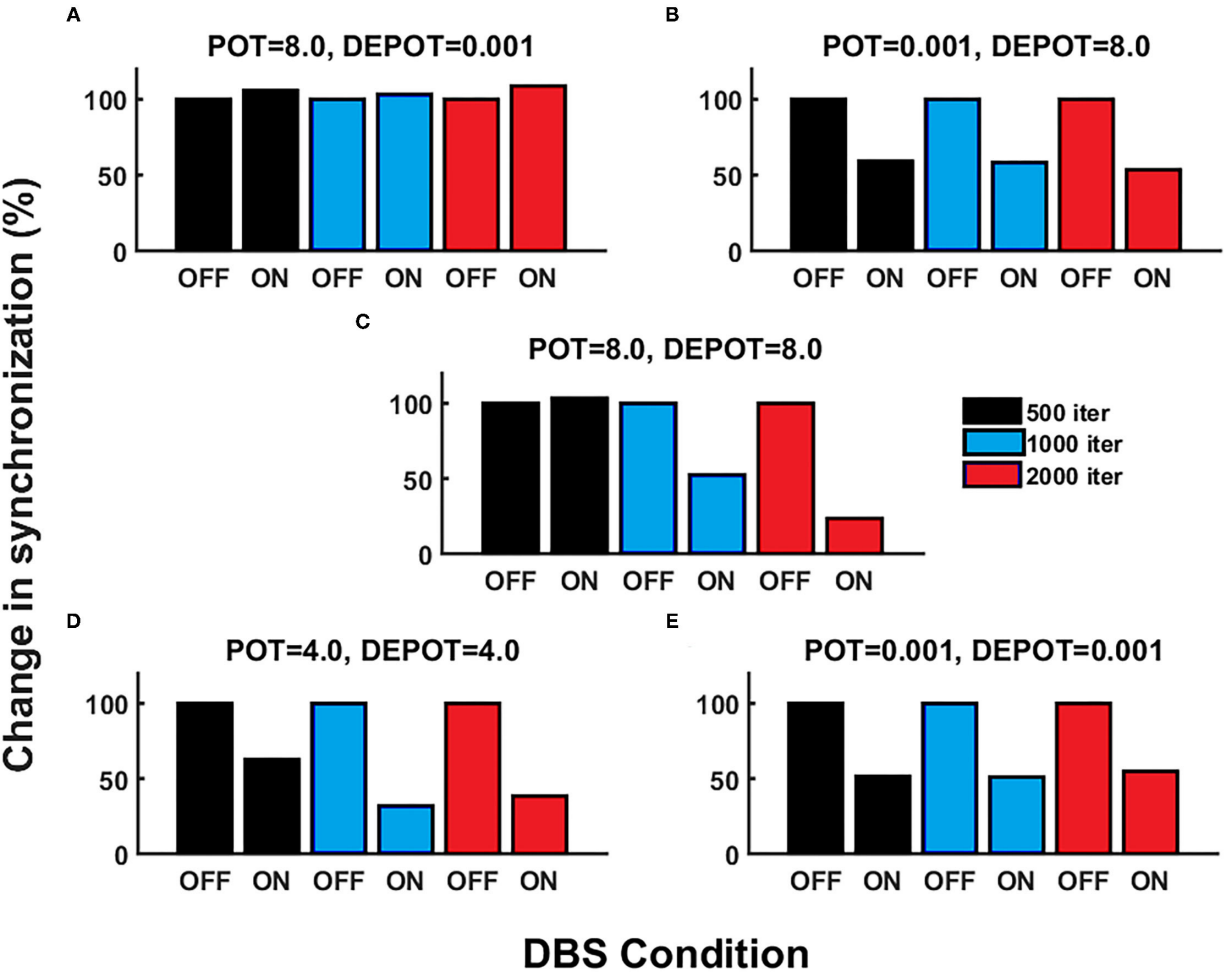
The simulation results [percentage change of the grand average PLV, the initial DBS OFF represents the baseline level (100%)] corresponding to the influence of stimulation duration (500, 1,000, and 2,000 iterations) in *de novo* DBS “patients”: unbalanced potentiation and depotentiation (high and low level) **(A,B)**; balance potentiation and depotentiation, i.e., equally high levels, **(C)**; equally medium levels **(D)**; and equally low levels **(E)**.

With a focus on longitudinal development under DBS input in *de novo* DBS “patients,” we again altered the level and balance of potentiation and depotentiation as depicted by Figures 3A,B, 4A,B. Strikingly, a stable high level of PLV was noticeable across a number of iterations (500, 1,000, and 2,000) (Figure 3A) and thus reflected the duration of DBS input, with a slight increase during DBS ON (Figure 4A) in the case of high potentiation and low depotentiation. A stable low level of PLV was noticeable across a number of iterations (500, 1,000, and 2,000) (Figure 3B) with a tendency to decrease during DBS OFF and DBS ON (Figure 4B) in the case of low potentiation and high depotentiation. The case of symmetry in the level of potentiation and depotentiation is depicted in Figures 3C–E, 4C–E. It is noticeable that for high levels of potentiation and depotentiation (8.0 and 8.0), PLV first increased and then showed a tendency to decrease in the transition from DBS OFF to DBS ON across a number of iterations (Figures 3C, 4C); in the case of middle values of potentiation and depotentiation (4.0 and 4.0), PLV first increased and then showed a tendency to decrease during DBS OFF while a tendency to decrease was observed during DBS ON across a number of iterations (Figures 3D, 4D); in the case of very low levels of potentiation and depotentiation (0.001 and 0.001), PLV showed low values with a tendency to decrease during DBS OFF and DBS ON across a number of iterations (Figures 3E, 4E).

## Discussion

This paper summarizes a computational modeling study in scenarios with different neuroplasticity distributions, reflecting virtual “patients” with different neurophysiological signatures. When a powerful treatment, such as deep brain stimulation gets introduced in a new patient, questions arise as to “Why there is no effect? Will there be an effect when I stimulate for a longer time? What happens when the DBS machinery fails or a planned interruption of the stimulation occurs?” In long-term patients on this neuromodulation treatment, the question comes up whether a break or time off the intervention could be planned, or whether a life-long dependence on this input is likely.

In line with existing neurobiological models and neurophysiological findings in various DBS-treated conditions, our study looks at synchronization (coupling) of oscillatory activity, assuming that too much synchronization in certain frequency bands (beta in PD, theta in dystonia, etc.) maintains the pathological state (rigidity, dystonic symptoms, obsessive compulsive behavior, etc.), whereas a reduction in synchronization reflects the leaving of the symptom-stabilizing state. The *in vivo* situation of a biological system of course is complex and the exact mechanism of action leading to symptom improvement initiated by disruption of the synchronization patterns through DBS remains unclear (46).

The adopted computational simulation approach is particularly advantageous with regards to avoiding the risks associated with turning on and off an implanted DBS device in patients suffering from neurological or psychiatric pathology and allowing flexibility in setting up the different plasticity scenarios that would not be accessible simultaneously under real (*in vivo*) patient conditions.

In the first part of our study, we simulated patients with DBS switched on long-term, and observed the effect of consecutive withdrawals and renewals of DBS. In the second part of the study, *de novo* “patients” (with no previous DBS treatment) received long-term DBS of various durations. On DBS treatment, the literature shows that certain symptoms seem to respond very quickly (tremor in PD) whereas others need longer, yet variable, time to allow the DBS effects on symptom alleviation to occur (dystonia) (9, 47). Particular patient profiles, that is, individual differences in baseline potentiation or depotentiation changes due to symptoms, have never been profiled and could provide information as to the potential response patients have to neuromodulation settings and treatment duration.

Our study introduces different neuroplasticity makeups, i.e., different levels and balances of potentiation and depotentiation. It assumes that this mirrors some real patient electrophysiological signatures. It is well-established that dystonia tends to show too much neuroplasticity, whereas it tends to be at normal levels in obsessive-compulsive disorders (OCD), and there is a lack of it in PD, Tourette Syndrome, bipolar disorder, and schizophrenia, as examples for abnormalities (48–50). These citations all represent group level studies, however, individual neuroplasticity levels are relevant for the personal effect of withdrawal or insertion of therapeutic input.

For the first part of the study, the simulations revealed that symmetry and asymmetry in the potentiation and depotentiation levels have a strong effect on the stability and level of synchronization patterns across the successive withdrawals and renewals of DBS input to the system. Our results reveal that, interestingly, a high level of potentiation in combination with a low level of depotentiation ensures the system is “stuck” in its current state (low mutability). Whether DBS is switched on or off does not affect the oscillatory state of the system. It remains stabilized at its observable high level of synchronization. On the contrary, when potentiation is low, independent of whether this is in combination with high or low depotentiation values, the oscillatory state of the system is highly mutable with a striking effect of switching DBS on or off. However, the effects of switching DBS on or off seem highly predictable. Intriguingly, when potentiation and depotentiation are balanced, both high or medium, the response to repeated insertions and withdrawals of DBS becomes less predictable and might suggest that they both counteract each other in line with a homeostatic regulation of the system, where a drive in one direction is counteracted *via* the other mechanism and driven into the opposite direction, with the goal of keeping the system in healthy boundaries. We conclude that the reaction of the oscillatory system highly depends on the neuroplasticity makeup of the “individual.” Let us assume that there are disorders with too much plasticity, i.e., too much potentiation: In this case, the reaction of the system to DBS withdrawal or reinsertion is almost ineffective. The system is stabilized in its current status. This situation resembles that of many DBS naïve dystonia patients, for example. The long time to respond to initial treatment in dystonia or the strong resistance to neuromodulation and to occupational training might be due to such a neurophysiological signature. In the low potentiation constellation, the oscillatory system instantly responds to being switched on or off. The system is highly mutable, a response well-known for major symptoms of PD and long-term DBS-treated dystonia patients who have low potentiation as a cardinal neurophysiological feature (9, 47). The change of the oscillatory system directly matches the ON/OFF state and is highly predictable. The most complicated constellation arises with balanced high or medium levels of potentiation and depotentiation. Unlike what happens in the high unbalanced potentiation situation, in this case, the system responds to insertion and withdrawal of DBS. However, the contradictory forces of neuroplasticity seem to intermingle and one might speculate that the homeostatic mechanisms come into play to keep the system within healthy boundaries. Here, the outcome of DBS OFF and ON scenarios becomes unpredictable. However, change happens, and this stands in contrast to the unbalanced high potentiation-low depotentiation scenario where the oscillatory system remains immutable and “stuck” in its current state.

In the second part of our study, the computational simulation mimicked a *de novo* “patient” who then received DBS for various durations (iterations), and consecutively withdrawal of DBS was simulated at different time points along this time axis. Again, as in the first part of the study, the outcome was highly dependent on the synaptic plasticity signature. In the unbalanced high potentiation-low depotentiation situation, the system over time remains immutable and “stuck” in its oscillatory state. DBS insertion is not able to produce change in the system, resembling the situation of therapy-resistant patients. As an example, naïve patients with dystonia are known for their high potentiation. The removal of high potentiation, allowing the system to become mutable, might drive the change toward the beneficial effects of DBS as suggested before (9). In the balanced high and medium potentiation-depotentiation scenario, we observe effects in a monophasic positive direction after insertion of DBS with a gradual decrease in coupling in the oscillatory system. Interestingly, however, upon withdrawal of DBS, a rebound occurs that exceeds the level of the initial coupling by far. This might resemble a situation of dramatic, sometimes life threatening worsening of symptoms in dystonia patients with accidental or planned DBS switch OFF (51). In a scenario where there is an unbalanced or balanced low potentiation, the response to DBS is present and in a positive direction, it appears that the system is mutable. Intriguingly, the oscillatory system reverts back to the DBS naïve state upon DBS withdrawal, but not to the 100% baseline level of the naïve system, in other words some of the DBS-induced effect seems to be stabilized despite a relative lack of potentiation. However, this is only a mild deviation from the naïve 100% value in the OFF state (before treatment or DBS input was initiated).

The computational simulation study shows the strong effect of the individual neuroplasticity makeup on the insertion and withdrawal of DBS as a therapeutic tool on the oscillatory system and thereby supposedly on disorder symptoms manifesting in brain coupling. How mutable a system is and thereby how effectively it responds to the treatment input might be linked to such individual signatures. Even in healthy people, neuroplasticity levels are variable and individual (52, 53). The meaning of such a marker setup or signature for personalized therapy and management of patients becomes clear by the usage of such computational approaches.

Limitations of the study or points to consider: This is a computational modeling and simulation approach and not *in vivo* data. However, in this limitation lies strength, because it is impossible, due to technical limitations, to obtain such data during acquisition of this type of neurophysiological recordings in real patients. Besides technical impossibility, the potential harm of switch OFF situations needs to be carefully considered by experienced clinicians who know the patients well. The second point to consider is that this modeling does not currently include the fact that the neuroplasticity itself is dependent on the oscillatory system and therefore will be dynamic over time. In other words, the results of the current study reflect the neuroplasticity conditions that once set up remain the same across iterations. As shown previously, the neuroplasticity is variable over time and also influences the switch OFF clinical outcome based on its potentiation as one form of neuroplasticity at that particular time point (9, 11, 12, 54). To explore the dynamics of the system would be a meaningful next study.

In conclusion, the electrophysiological signature has a profound impact on the effects of an intervention, such as DBS on the system, and can be used in future to narrow down potential outcomes in specific scenarios.

## Data Availability Statement

The raw data supporting the conclusions of this article will be made available by the authors, without undue reservation.

## Author Contributions

All authors listed have made a substantial, direct, and intellectual contribution to the work and approved it for publication.

## Conflict of Interest

The authors declare that the research was conducted in the absence of any commercial or financial relationships that could be construed as a potential conflict of interest.

## Publisher's Note

All claims expressed in this article are solely those of the authors and do not necessarily represent those of their affiliated organizations, or those of the publisher, the editors and the reviewers. Any product that may be evaluated in this article, or claim that may be made by its manufacturer, is not guaranteed or endorsed by the publisher.
